# Development and Validation of Prediction Models for the Prognosis of Clear Cell Adenocarcinoma of the Cervix: A Population‐Based Cohort Study

**DOI:** 10.1002/cam4.71585

**Published:** 2026-01-26

**Authors:** Lijun Chen, Linying Liu, Feishuang Lin, Yixin Fu, Lin Yang, Yang Sun

**Affiliations:** ^1^ Department of Gynecology Clinical Oncology School of Fujian Medical University, Fujian Cancer Hospital Fuzhou Fujian China

**Keywords:** cancer‐specific survival, clear cell adenocarcinoma of the cervix, nomogram, overall survival, SEER

## Abstract

**Aims:**

Clear cell adenocarcinoma of the cervix (CCAC) is a rare and aggressive malignancy with poor prognosis. This study aimed to develop and validate nomograms and risk‐stratification scores for predicting overall survival (OS) and cancer‐specific survival (CSS) in CCAC patients.

**Methods:**

Data from 429 CCAC patients were extracted from the Surveillance, Epidemiology, and End Results (SEER) database (2000–2019). Patients were randomly assigned to training and validation sets. Cox regression analysis identified five independent prognostic factors for OS and CSS, which were used to construct nomograms for predicting 1‐, 3‐, and 5‐year OS and CSS. The models were evaluated using receiver operating characteristic (AUC) analysis, calibration curves, and decision curve analysis (DCA). The clinical utility of the nomograms was compared with the 2018 FIGO Stage System using C‐index, NRI, and IDI. Patients were stratified into low‐ and high‐risk groups based on predicted risk scores, and Kaplan–Meier survival analysis was performed.

**Results:**

Multivariate Cox regression identified age at diagnosis, tumor size, and surgery as independent prognostic factors for both OS and CSS, while chemotherapy and radiotherapy were specifically associated with OS and CSS. The C‐index for OS and CSS in the training set was 0.83 and 0.84, respectively. The AUC for 1‐, 3‐, and 5‐year OS and CSS in the training set was 0.95, 0.95, and 0.88, respectively, with similar results in the validation set. Calibration curves showed good agreement between predicted and actual outcomes. DCA, NRI, and IDI analyses indicated that the nomogram outperformed the 2018 FIGO Stage System. Survival analysis revealed that high‐risk patients had worse prognosis compared to low‐risk patients.

**Conclusion:**

This study developed and validated nomograms for predicting OS and CSS in CCAC patients using SEER data. These models offer a more accurate prognostic tool, enhancing clinical decision‐making and enabling individualized treatment planning.

## 1 Introduction

Clear cell adenocarcinoma of the cervix (CCAC) is a rare, HPV‐independent, and aggressive neoplasm, accounting for only 4%–9% of all adenocarcinomas of the uterine cervix [1]. The etiology and pathogenesis of CCAC remain poorly understood, but it has been associated with intrauterine exposure to diethylstilbestrol (DES), which was prescribed to pregnant women between the 1940s and 1970s [2, 3, 4]. DES‐exposed individuals tend to present at a younger age, with a peak incidence around 19 years [5, 6]. Following the ban on DES in the 1970s, a growing number of non‐DES‐exposed CCAC have been reported [7, 8, 9, 10].

Due to its low incidence, clear cell adenocarcinoma of the cervix (CCAC) remains under‐researched, with most studies being small and retrospective [8, 11, 12, 13]. Li et al. were the first to systematically explore the clinical features, treatment patterns, and prognosis of CCAC using the Surveillance, Epidemiology, and End Results (SEER) database [14]. Their findings revealed that CCAC patients had a poorer prognosis compared to other histological subtypes, such as squamous cell carcinoma, usual type adenocarcinoma, and adenosquamous carcinoma, after excluding those with metastatic disease. Despite the rarity of CCAC, several prognostic variables have been identified, including age at diagnosis, tumor stage, tumor size, and lymph node metastasis, etc. [8, 13, 14]. Although the FIGO and AJCC staging systems are widely used to predict survival in cervical cancer, they primarily rely on tumor size, local extension, and lymph node involvement. For rare subtypes like CCAC, these factors alone may not capture the full spectrum of prognostic influences. CCAC exhibits unique clinical and biological characteristics, including age distribution, DES exposure history, and potentially distinct molecular profiles [11, 12, 15], that may meaningfully impact outcomes. However, conventional staging systems provide only broad risk categories and limited individualized prognostic information.

Therefore, there is a clear need for a more tailored prognostic model that integrates multiple clinical variables to better stratify patients and guide individualized treatment strategies. In this study, we utilized the SEER database to construct nomograms for predicting overall survival (OS) and cancer‐specific survival (CSS) in patients with CCAC, providing a more comprehensive and personalized prognostic tool for clinical decision‐making.

## 2 Method

### 2.1 Study Design

This study is a retrospective cohort analysis based on the SEER Program of the National Cancer Institute (NCI) database (http://www.seer.cancer.gov). Data were extracted from the SEER database, encompassing the years 1975 to 2020 and including patients diagnosed with CCAC. It aimed at identifying prognostic factors for CCAC and developing nomograms to predict OS and CSS. As the clinical information and data used in this SEER study were downloaded from a public database, ethical approval and informed consent were waived.

### 2.2 Data Source, Patient Selection and Variables

Data retrieval was conducted online using SEER*Stat 8.4.0 software. Eligible patients were identified from “SEER Research Plus Data, 17 Registries, Nov 2021 sub ([2000‐2019])”. “Site and Morphology, Behavior recode for analysis” was limited to “Malignant”, and “Site and Morphology, Site recode ICD‐O‐3/WHO 2008” was limited to “Cervix Uteri”. Inclusion criteria include (1) histology types based on International Classification of Diseases (ICD)‐10 morphology codes: CCAC (8310), (2) patients with pathological diagnosis. Exclusion criteria encompass (1) more than one primary tumor cases, (2) follow up information less than 1 month. The selection process is shown in Figure S1. There were 429 cases and 424 cases for OS analysis and CSS analysis, respectively. The follow‐up ended on 11 September 2024.

The clinicopathological variables included age at diagnosis, year of diagnosis, race, marital status, histology grade, 2018 FIGO stage, TNM stage, tumor size, lymph node metastasis, treatment strategy (surgery, radiation, chemotherapy), vital status, cause of death, and survival times.

### 2.3 Statistical Analysis

All statistical analyses were performed using R software version 4.2.2. The eligible patients were divided into training and validation groups according to the ratio of 7:3. Continuous variables (age) were expressed under the form of mean and standard deviation or median and interquartile range, and were tested by independent variance *t*‐test or Mann–Whitney *U* test between the two groups. Count data were expressed by case frequency (%), and were compared by chi‐square test or Fisher exact test between groups. Cox proportional hazards regression analysis was performed on the training set to identify independent factors associated with OS and CSS. The impact of these factors on OS and CSS was assessed using hazard ratios (HR) and 95% confidence intervals (CI), with a *p* < 0.05 considered statistically significant.

Two nomograms were constructed to predict 1‐, 3‐, and 5‐year OS and CSS, integrating the independent prognostic factors identified in the multivariate cox analysis. The predictability of the models was assessed by area under the curve of receiver operating characteristic (AUC) value and concordance index (C‐index), of which the values over 0.7 indicated good predictability. We also performed internal validation on the training cohort using 1000 bootstrap resampling iterations and reported the average AUCs with 95% confidence intervals derived from the bootstrap distribution. Calibration curves were employed to compare the difference degree between the predicted and actual risks, and the time‐dependent Brier scores (IBS) were calculated to provide a quantitative measure of calibration. Decision curve analysis (DCA) was drawn to evaluate the clinical benefit and utility of the predictive models. Moreover, as the net reclassification index (NRI) only considers the improvement when a specific cutoff point is set and integrated discrimination improvement (IDI) inspects the overall improved performance of the model, both methods were used to evaluate the clinical benefits and utility of the nomogram model and the 2018 FIGO stage system. Furthermore, we divided patients of each cohort into low‐, middle‐, and high‐risk levels according to the model‐predicted risk score using X‐tile. Survival differences were evaluated using the Kaplan–Meier (K–M) plots and Log‐rank test, with a significance level set at *p* < 0.05.

## 3 Result

### 3.1 Baseline Characteristics

The detailed baseline clinical characteristics of all the patients are presented in Table 1. A total of 429 patients were included in the OS analysis, with 301 randomly assigned to the training set and the remaining to the validation set. For the CSS analysis, 424 patients were included, with 300 in the training cohort and 124 in the validation cohort. No significant differences were observed between the variables in the training and validation sets (*p* > 0.05), indicating successful randomization and comparability of the groups.

**TABLE 1:** Demographics and clinicopathologic characteristics of SEER patients with CCAC.

Variables	Level	OS				CSS			
		Overall (*n* = 429)	Training set (*n* = 301)	Validation set (*n* = 128)	*p*	Overall (*n* = 424)	Training set (*n* = 300)	Validation set (*n* = 124)	*p*
Age (median [IQR])		57.00 [42.00, 70.00]	57.00 [43.00, 70.00]	56.50 [42.00, 68.25]	0.569	56.50 [42.00, 69.25]	56.50 [42.75, 70.00]	56.50 [42.00, 68.25]	0.524
Race, *n* (%)	White	332 (77.4)	234 (77.7)	98 (76.6)	0.91	328 (77.4)	230 (76.7)	98 (79.0)	0.624
	Black	51 (11.9)	36 (12.0)	15 (11.7)		51 (12.0)	39 (13.0)	12 (9.7)	
	Other	46 (10.7)	31 (10.3)	15 (11.7)		45 (10.6)	31 (10.3)	14 (11.3)	
Marital status, *n* (%)	Single	135 (31.5)	96 (31.9)	39 (30.5)	0.527	134 (31.6)	98 (32.7)	36 (29.0)	0.765
	Married	171 (39.9)	115 (38.2)	56 (43.8)		168 (39.6)	117 (39.0)	51 (41.1)	
	Other	123 (28.7)	90 (29.9)	33 (25.8)		122 (28.8)	85 (28.3)	37 (29.8)	
Grade, *n* (%)	Well‐moderate	43 (10.0)	29 (9.6)	14 (10.9)	0.589	43 (10.1)	29 (9.7)	14 (11.3)	0.642
	Poor‐undifferented	216 (50.3)	148 (49.2)	68 (53.1)		213 (50.2)	148 (49.3)	65 (52.4)	
	Unknown	170 (39.6)	124 (41.2)	46 (35.9)		168 (39.6)	123 (41.0)	45 (36.3)	
2018 FIGO stage, *n* (%)	I	172 (40.1)	123 (40.9)	49 (38.3)	0.402	171 (40.3)	127 (42.3)	44 (35.5)	0.118
	II	55 (12.8)	39 (13.0)	16 (12.5)		55 (13.0)	32 (10.7)	23 (18.5)	
	III	97 (22.6)	71 (23.6)	26 (20.3)		95 (22.4)	70 (23.3)	25 (20.2)	
	IV	80 (18.6)	49 (16.3)	31 (24.2)		79 (18.6)	52 (17.3)	27 (21.8)	
	Unknown	25 (5.8)	19 (6.3)	6 (4.7)		24 (5.7)	19 (6.3)	5 (4.0)	
T, *n* (%)	T1	223 (52.0)	165 (54.8)	58 (45.3)	0.071	222 (52.4)	164 (54.7)	58 (46.8)	0.093
	T2	114 (26.6)	81 (26.9)	33 (25.8)		114 (26.9)	77 (25.7)	37 (29.8)	
	T3	52 (12.1)	33 (11.0)	19 (14.8)		49 (11.6)	37 (12.3)	12 (9.7)	
	T4	14 (3.3)	6 (2.0)	8 (6.2)		14 (3.3)	6 (2.0)	8 (6.5)	
	Unknown	26 (6.1)	16 (5.3)	10 (7.8)		25 (5.9)	16 (5.3)	9 (7.3)	
N, *n* (%)	Negative	257 (59.9)	182 (60.5)	75 (58.6)	0.916	255 (60.1)	181 (60.3)	74 (59.7)	0.967
	Positive	128 (29.8)	88 (29.2)	40 (31.2)		127 (30.0)	90 (30.0)	37 (29.8)	
	Unknown	44 (10.3)	31 (10.3)	13 (10.2)		42 (9.9)	29 (9.7)	13 (10.5)	
M, *n* (%)	No	355 (82.8)	255 (84.7)	100 (78.1)	0.13	351 (82.8)	252 (84.0)	99 (79.8)	0.373
	Yes	74 (17.2)	46 (15.3)	28 (21.9)		73 (17.2)	48 (16.0)	25 (20.2)	
Tumor size, *n* (%)	≤ 40 mm	155 (36.1)	111 (36.9)	44 (34.4)	0.88	155 (36.6)	114 (38.0)	41 (33.1)	0.522
	> 40 mm	152 (35.4)	105 (34.9)	47 (36.7)		150 (35.4)	106 (35.3)	44 (35.5)	
	Unknown	122 (28.4)	85 (28.2)	37 (28.9)		119 (28.1)	80 (26.7)	39 (31.5)	
Surgery, *n* (%)	Yes	261 (60.8)	182 (60.5)	79 (61.7)	0.892	259 (61.1)	180 (60.0)	79 (63.7)	0.546
	No/Unknown	168 (39.2)	119 (39.5)	49 (38.3)		165 (38.9)	120 (40.0)	45 (36.3)	
Radiotherapy, *n* (%)	Yes	269 (62.7)	189 (62.8)	80 (62.5)	1	265 (62.5)	184 (61.3)	81 (65.3)	0.508
	No/Unknown	160 (37.3)	112 (37.2)	48 (37.5)		159 (37.5)	116 (38.7)	43 (34.7)	
Chemotherapy, *n* (%)	Yes	226 (52.7)	155 (51.5)	71 (55.5)	0.517	224 (52.8)	156 (52.0)	68 (54.8)	0.67
	No/Unknown	203 (47.3)	146 (48.5)	57 (44.5)		200 (47.2)	144 (48.0)	56 (45.2)	

### 3.2 Nomogram Variable Screening

As shown in Table 2, univariate Cox regression analysis in the training set showed 12 variables were significantly associated with OS, including age at diagnosis, race, marital status, differentiation, tumor size, TMN stages, FIGO stage, surgery, chemotherapy, and radiotherapy. Multivariate Cox regression analysis showed 5 variables (age, FIGO stage, tumor size, surgery, and chemotherapy) were independent prognostic factors in patients with clear cell carcinoma.

**TABLE 2:** Univariate and multivariate analysis for OS and CSS in training cohort.

Variables		OS				CSS			
		Univariate		Multivariate		Univariate		Multivariate	
		HR (95% CI)	*p*	HR (95% CI)	*p*	HR (95% CI)	*p*	HR (95% CI)	*p*
Age		1.032 (1.021–1.042)	**< 0.001**	1.036 (1.022–1.049)	**< 0.001**	1.022 (1.012–1.033)	**< 0.001**	1.026 (1.011–1.041)	**0.001**
Race	White								
	Black	1.683 (1.073–2.639)	**0.023**	1.169 (0.691–1.978)	0.561	1.554 (0.955–2.527)	0.076		
	Other	0.549 (0.287–1.049)	0.069	0.689 (0.337–1.406)	0.306	0.594 (0.288–1.226)	0.159		
Marital status	Single								
	Married	1.271 (0.826–1.956)	0.275	0.669 (0.410–1.094)	0.110	1.179 (0.743–1.872)	0.485	0.672 (0.399–1.130)	0.133
	Other	1.968 (1.277–3.031)	**0.002**	0.626 (0.361–1.087)	0.096	1.768 (1.101–2.838)	**0.018**	0.72 (0.408–1.270)	0.256
Grade	Well‐moderate								
	Poor‐undifferented	2.125 (1.060–4.260)	**0.034**	1.145 (0.537–2.438)	0.726	2.11 (0.961–4.630)	0.063	1.311 (0.545–3.155)	0.546
	Unknown	2.905 (1.442–5.853)	**0.003**	1.316 (0.608–2.849)	0.486	2.878 (1.303–6.353)	**0.009**	1.442 (0.595–3.496)	0.418
2018 FIGO stage	I								
	II	3.597 (2.068–6.257)	**< 0.001**	2.283 (0.963–5.412)	**0.061**	5.22 (2.548–10.695)	**< 0.001**	4.905 (1.732–13.896)	**0.003**
	III	3.81 (2.347–6.186)	**< 0.001**	3.189 (1.350–7.537)	**0.008**	6.089 (3.311–11.198)		6.255 (2.428–16.116)	**< 0.001**
	IV	11.489 (7.009–18.834)	**< 0.001**	9.21 (1.120–75.740)	**0.039**	19.794 (10.791–36.306)	**< 0.001**	4.938 (0.540–45.152)	0.157
	Unknown	3.554 (1.810–6.978)	**< 0.001**	1.451 (0.406–5.189)	0.567	6.315 (2.833–14.077)	**< 0.001**	4.55 (1.078–19.198)	0.039
T	T1								
	T2	3.031 (2.035–4.515)	**< 0.001**	1.549 (0.804–2.986)	0.191	3.336 (2.109–5.279)	**< 0.001**	0.908 (0.463–1.779)	0.778
	T3	5.181 (3.196–8.397)	**< 0.001**	0.863 (0.427–1.744)	0.682	6.175 (3.684–10.352)	**< 0.001**	0.64 (0.318–1.288)	0.211
	T4	7.084 (2.794–17.965)	**< 0.001**	0.614 (0.139–2.705)	0.519	9.399 (3.648–24.212)	**< 0.001**	3.095 (0.573–16.716)	0.189
	Unknown	5.214 (2.765–9.832)	**< 0.001**	1.297 (0.583–2.888)	0.524	6.195 (3.053–12.571)	**< 0.001**	0.928 (0.358–2.403)	0.877
N	Negative								
	Positive	2.615 (1.819–3.758)	**< 0.001**	0.976 (0.472–2.016)	0.947	3.383 (2.264–5.054)	**< 0.001**	0.898 (0.451–1.788)	0.76
	Unknown	3.051 (1.897–4.906)	**< 0.001**	0.656 (0.258–1.669)	0.376	3.733 (2.160–6.449)	**< 0.001**	0.597 (0.232–1.535)	0.284
M	No								
	Yes	5.381 (3.667–7.895)	**< 0.001**	1.148 (0.161–8.206)	0.890	6.402 (4.265–9.609)	**< 0.001**	3.167 (0.416–24.121)	0.266
Tumor size	≤ 40 mm								
	> 40 mm	3.032 (1.956–4.700)	**< 0.001**	2.177 (1.302–3.642)	**0.003**	3.682 (2.228–6.082)	**< 0.001**	2.172 (1.250–3.774)	**0.006**
	Unknown	3.243 (2.097–5.017)	**< 0.001**	1.954 (1.160–3.292)	**0.012**	3.91 (2.331–6.562)	**< 0.001**	1.69 (0.934–3.057)	**0.083**
Surgery	Yes								
	No/Unknown	4.919 (3.495–6.924)	**< 0.001**	3.397 (2.155–5.355)	**< 0.001**	6.513 (4.364–9.720)	**< 0.001**	4.41 (2.656–7.322)	**< 0.001**
Radiotherapy	Yes								
	No/Unknown	0.595 (0.414–0.853)	**0.005**	1.37 (0.850–2.209)	0.196	0.613 (0.412–0.913)	**0.016**	2.131 (1.273–3.567)	**0.004**
Chemotherapy	Yes								
	No/Unknown	0.65 (0.464–0.909)	0.012	2.107 (1.301–3.414)	**0.002**	0.733 (0.505–1.064)	0.103		

*Note:* Bold values indicate statistical significance values *p* < 0.05.

For the grouping status of CSS, univariate Cox regression analysis demonstrated that 10 variables were CSS‐related prognostic factors, including age at diagnosis, marital status, differentiation, tumor size, TMN stages, FIGO stage, surgery, and radiotherapy. Among these variables, age at diagnosis, tumor size, FIGO stage, surgery, and radiotherapy were found to be significant predictors (Table 2).

### 3.3 Nomogram Construction and Validation

**FIGURE 1:**
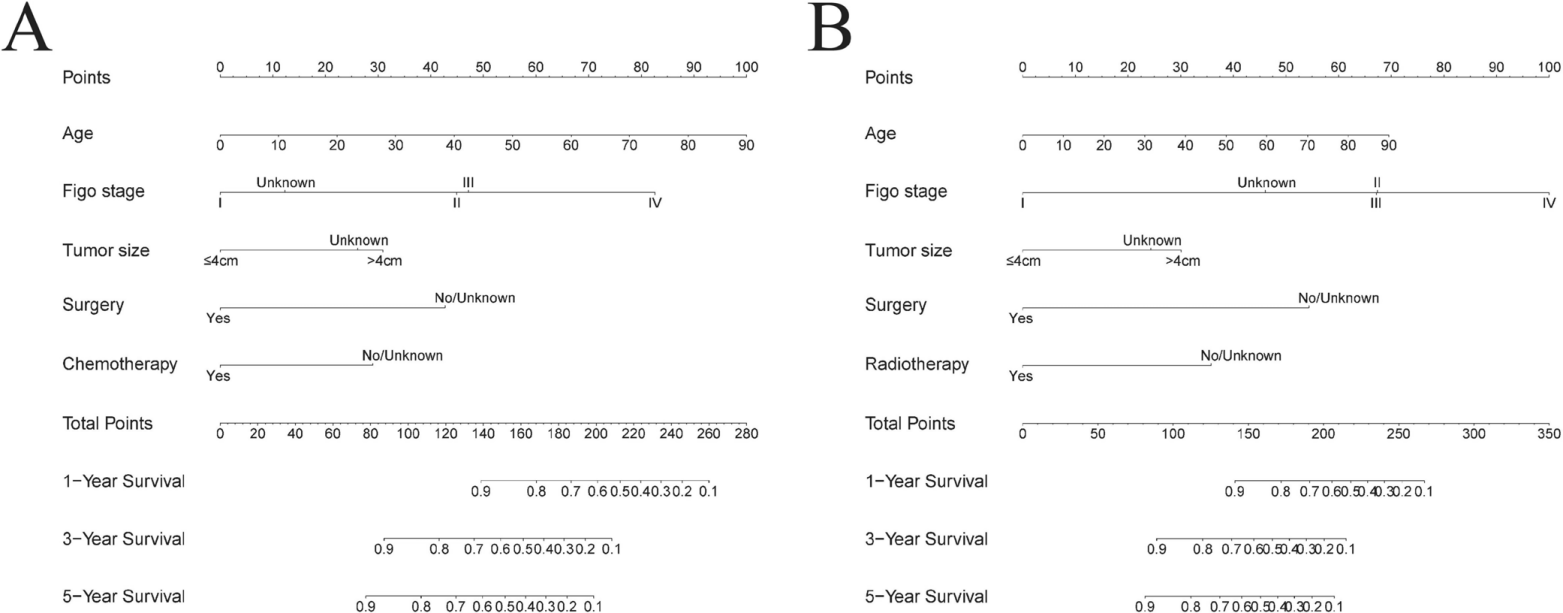
Nomograms for predicting the 1‐, 3‐, and 5‐year OS (A) and CSS (B) in CCAC patients.

**FIGURE 2:**
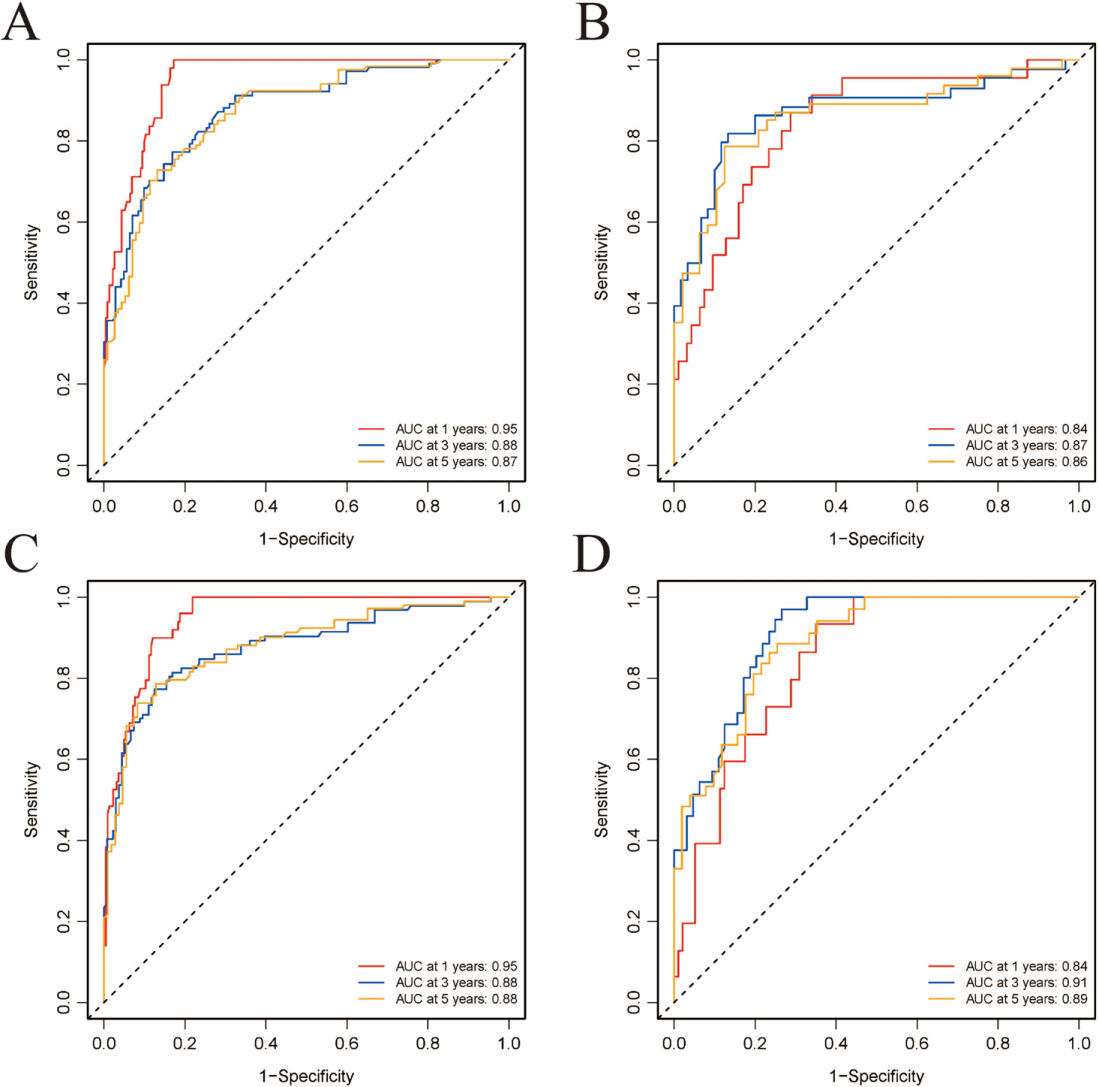
ROC curve analysis for 1‐, 3‐, and 5‐year survival of nomogram. (A) OS in the training set, (B) OS in the validation set, (C) CSS in the training set, (D) CSS in the validation set.

Based on these statistically significant variables, we developed two nomogram models to predict 1‐year, 3‐year, and 5‐year OS and CSS for CCAC patients using the training cohort (Figure 1). The C‐index for OS and CSS predictions was 0.83 (95% CI = 0.80–0.87) and 0.84 (95% CI = 0.81–0.88) in the training set, respectively. Both nomograms demonstrated good discrimination in both training and validation sets, with AUCs of 0.95, 0.95, and 0.88 for 1‐, 3‐, and 5‐year OS in the training set (Figure 2A), and 0.83, 0.83, and 0.88 for 1‐, 3‐, and 5‐year OS in the validation set (Figure 2B). After 1000 bootstrap resampling iterations, the model demonstrated consistently high discrimination ability. The bootstrap‐corrected mean AUCs for 1‐, 3‐, and 5‐year survival were 0.95 (95% CI: 0.92–0.97), 0.88 (95% CI: 0.84–0.92), and 0.88 (95% CI: 0.83–0.92), respectively. For CSS analysis, the AUCs for 1‐, 3‐, and 5‐year survival in the training cohort were 0.95, 0.95, and 0.88, respectively (Figure 2C), while in the validation cohort, the AUCs were 0.92 at 1 and 3 years, and 0.94 at 5 years (Figure 2D). The bootstrap‐corrected mean AUCs for 1‐, 3‐, and 5‐year survival were 0.95 (95% CI: 0.92–0.97), 0.88 (95% CI: 0.83–0.93), and 0.88 (95% CI: 0.84–0.93), respectively. The AUCs of both OS and CSS predictive models were higher than that of the FIGO model (Figure S2).

**FIGURE 3:**
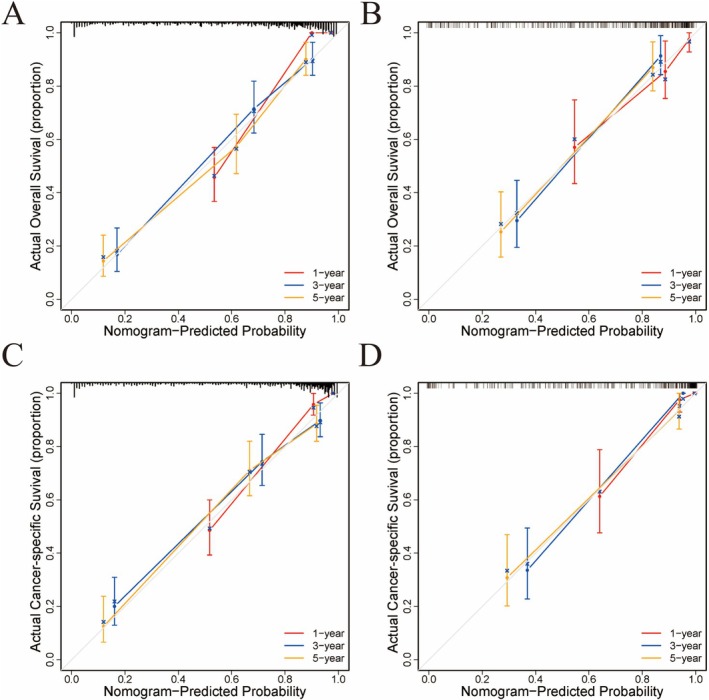
Calibration curves for 1‐, 3‐, and 5‐year survival. (A) OS in the training set, (B) OS in the validation set, (C) CSS in the training set, (D) CSS in the validation set.

**FIGURE 4:**
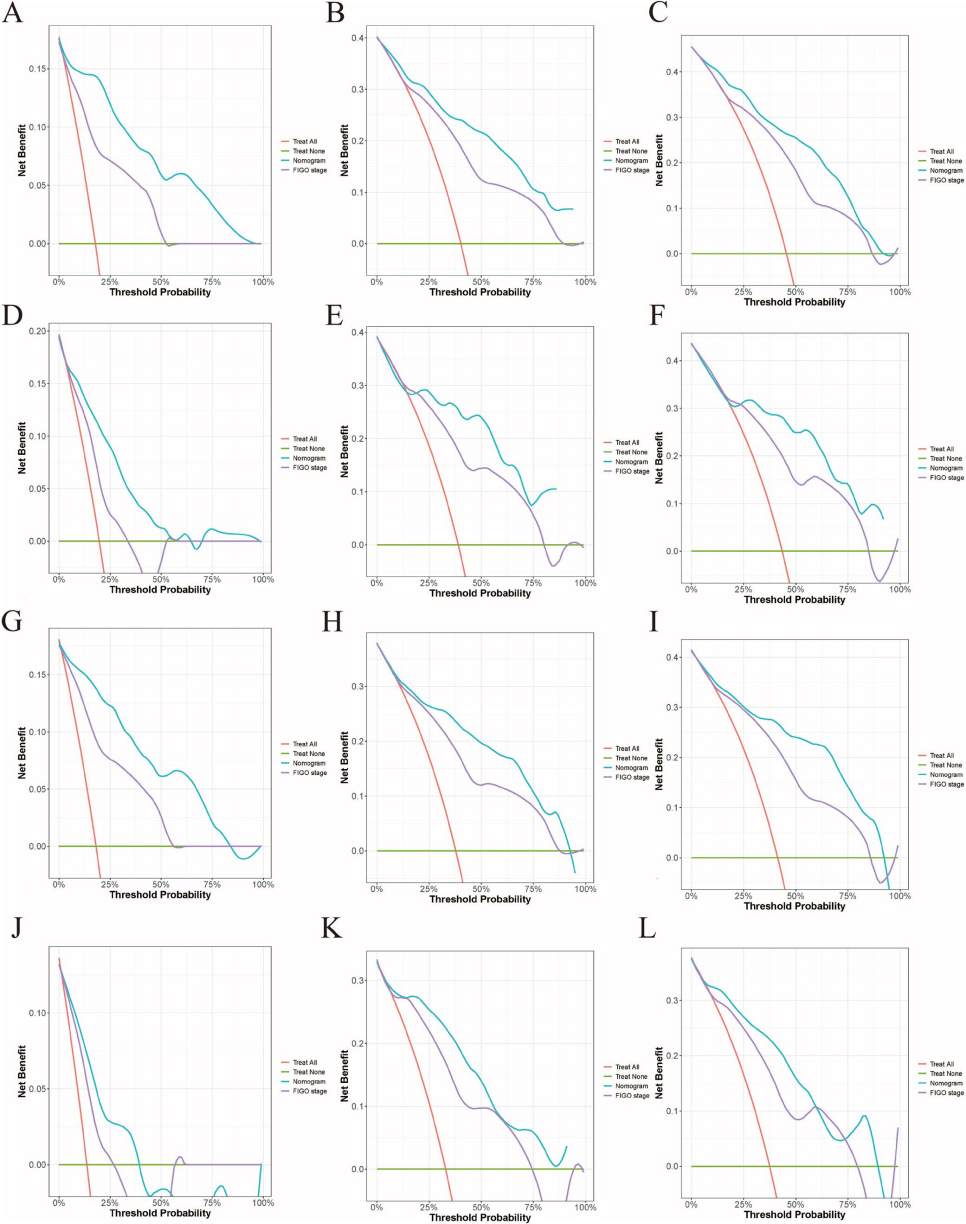
Decision curves for predicting survival. (A–C) 1‐, 3‐, and 5‐ OS nomogram in training set, (D–F) 1‐, 3‐, and 5‐ OS nomogram in validation set, (G–I) 1‐, 3‐, and 5‐ CSS nomogram in training set, (J–L) 1‐, 3‐, and 5‐year CSS nomogram in validation set.

The calibration curves for 1‐, 3‐, and 5‐year OS and CSS survival probabilities demonstrated good agreement between the predicted probabilities and actual outcomes in both the training and validation sets (Figure 3). The IBS of 0.141 for the OS predictive model and 0.134 for the CSS predictive model. These findings suggest that the nomograms can accurately predict survival in patients with clear cell carcinoma. Furthermore, the DCA for 1‐, 3‐, and 5‐year OS and CSS in both the training and validation cohorts indicated better clinical applicability of the two prediction models than the 2018 FIGO stage system (Figure 4).

### 3.4 Clinical Value Comparison Between Nomograms and FIGO Stage System

To evaluate the added value of the nomograms, we compared their predictive performance with the FIGO 2018 staging system using C‐index, NRI, and IDI. The C‐index was higher for the nomograms than for FIGO staging alone. In the validation cohort, the C‐index for the OS and CSS nomograms was 0.81 (95% CI: 0.75–0.87) and 0.877 (95% CI: 0.809–0.907), respectively, compared with 0.73 (95% CI: 0.66–0.79) and 0.778 (95% CI: 0.722–0.834) for FIGO stage, indicating that the nomograms have superior discriminative accuracy. The NRI and IDI values further confirmed that the nomograms improved risk classification over FIGO staging. For example, the 5‐year OS NRI in the validation set was 0.287 (95% CI: 0.069–0.561), indicating that the nomogram more accurately reclassified patients into appropriate risk categories. The IDI value of 5‐year CSS was 0.154 (95% CI = 0.072–0.276, *p* < 0.001) in the validation cohort, indicating that the nomogram improved the overall discrimination ability by better distinguishing patients with different survival probabilities. These findings were consistent for 1‐ and 3‐year survival as well, indicating that the nomogram predicted prognosis with greater accuracy than the 2018 FIGO staging system. Additional details are provided in Table 3.

**TABLE 3:** Comparison of nomogram model and 2018 FIGO stage system for estimating the OS and CSS of CCAC patients.

Index		Training cohort			Validation cohort		
		Estimate	95% CI	*p*	Estimate	95% CI	*p*
OS	NRI (vs. 2018FIGO stage system)						
	For 1‐year	0.559	0.394–0.708	**< 0.001**	0.355	0.123–0.588	**0.002**
	For 3‐year	0.44	0.305–0.573	**< 0.001**	0.249	0.112–0.559	**0.004**
	For 5‐year	0.456	0.3–0.588	**< 0.001**	0.287	0.094–0.566	**0.002**
	IDI (vs. 2018FIGO stage system)						
	For 1‐year	0.15	0.081–0.238	**< 0.001**	0.175	0.072–0.32	**< 0.001**
	For 3‐year	0.157	0.085–0.240	**< 0.001**	0.165	0.008–0.272	**0.002**
	For 5‐year	0.163	0.084–0.247	**< 0.001**	0.154	0.072–0.276	**< 0.001**
	C‐index						
	The nomogram	0.83	0.80–0.87		0.81	0.75–0.87	
	2018FIGO stage system	0.74	0.70–0.78		0.73	0.66–0.79	
CSS	NRI (vs. 2018FIGO stage system)						
	For 1‐year	0.634	0.453–0.724	**< 0.001**	0.327	0.071–0.643	**0.026**
	For 3‐year	0.449	0.3–0.607	**0.002**	0.445	0.096–0.629	**0.022**
	For 5‐year	0.441	0.285–0.600	**< 0.001**	0.476	0.113–0.653	**0.02**
	IDI (vs. 2018FIGO stage system)						
	For 1‐year	0.178	0.104–0.271	**< 0.001**	0.1	0.018–0.281	**0.006**
	For 3‐year	0.163	0.087–0.25	**< 0.001**	0.156	0.027–0.297	**0.022**
	For 5‐year	0.148	0.075–0.238	**< 0.001**	0.161	0.045–0.321	**0.012**
	C‐Index						
	The nomogram	0.842	0.806–0.877		0.877	0.809–0.907	
	2018FIGO stage system	0.773	0.734–0.812		0.778	0.722–0.834	

*Note:* Bold values indicate statistical significance values *p* < 0.05.

Abbreviations: IDI, integrated discrimination improvement; NRI, net reclassification improvement.

### 3.5 Risk Stratification for CCAC

**FIGURE 5:**
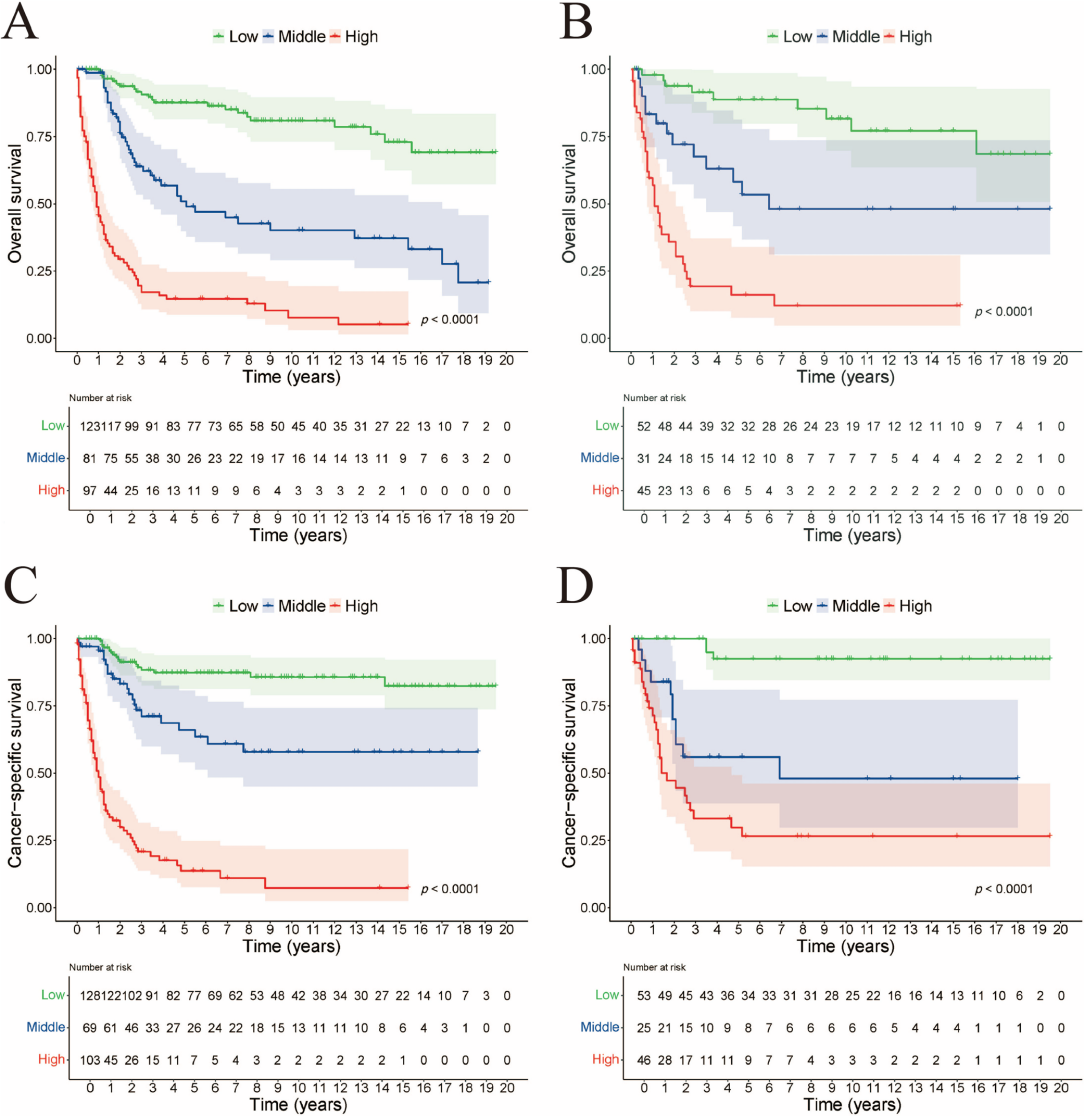
Kaplan–Meier curves of CCAC patients with different risks stratified by the nomogram. (A) OS in training set, (B) OS in validation set, (C) CSS in training set, (D) CSS in validation set.

We calculated total scores based on the nomogram for risk stratification. The patient was therefore classified into two risk groups (low‐risk and high‐risk) based on the median risk score. The Kaplan–Meier survival analysis revealed that the low‐risk group had a better OS and CSS than the high‐risk group (Figure 5). Both results were confirmed in the validation cohort.

## 4 Discussion

As a sub‐type of cervical cancer, CCAC is characterized by aggressive behavior and poor clinical outcomes. Despite the widespread use of the FIGO staging system and AJCC for cervical cancer, these do not provide clear guidelines for rare and poorly prognostic subtypes such as CCAC. Therefore, this study aimed to develop practical nomograms to predict survival outcomes in CCAC patients. We identified key prognostic factors, including age, tumor size, FIGO stage, surgery, and chemotherapy (radiotherapy), which were associated with OS and CSS. The developed nomograms for OS and CSS demonstrated superior prediction ability compared to the FIGO staging system. This study offers a more accurate survival prediction tool for clinical use in managing CCAC patients.

One of the strengths of this study is the use of a large SEER database, which covers a substantial number of CCAC patients and provides a reliable statistical basis for the model's construction. In clinical practice, many challenges prevent the establishment of effective treatment plans for rare cancer types. The SEER database has been widely used in research on rare cancers, such as neuroendocrine carcinoma of the cervix and primary vaginal cancer [16, 17, 18]. Given the lack of research on predicting OS and CSS in CCAC patients, it is essential to identify critical prognostic factors that can guide clinical decision‐making.

Through Cox regression analysis, we identified five independent prognostic factors, which were incorporated into a nomogram. This process ensures that the constructed models are both statistically significant and reflect the clinical characteristics of CCAC patients. Age was recognized as an important feature in disease onset [6], with a bimodal distribution in CCAC incidence, particularly in non‐DSE‐related cases. Recent studies have reported that non‐DSE‐related CCAC tends to present at a younger age [19, 20, 21]. However, previous real‐world studies have not found a clear correlation between age and prognosis, possibly due to small sample sizes [13]. In contrast, our study revealed that older age was significantly associated with OS and CSS, which aligns with Li's study, which categorized age and found that patients over 50 years had poorer OS [14]. This finding also supports Huo et al. work, which reported that mortality in CCAC increases with age [6]. Therefore, special attention should be given to elderly CCAC patients, with early interventions being crucial for improving their prognosis.

Our study revealed that CCAC patients with advanced FIGO stage and larger tumor size (> 4 cm) had worse OS and CSS, consistent with prior study [9]. Stolnicu et al. published a study with the largest cohort of CCAC patients to date, finding that FIGO stage impacts recurrence‐free survival, while OS was influenced by recurrence [13]. Liu et al. also demonstrated that early stage (IB‐IIA) patients had significantly better 5–year progression‐free survival (PFS) and OS compared to advanced‐stage patients (IIB‐IIIC) [8]. However, due to the lack of recurrence information in the SEER database, and the maximum sample size of the prior study was only 58 cases, it is challenging to assess the role of recurrence on OS and CSS. Larger studies with more detailed recurrence data are needed to further investigate this relationship. Tumor size was also a key predictor of outcomes. Hanselaar et al. reported that tumor size (> 4 cm) was a negative prognostic factor for CCAC [5]. However, Liu et al. found tumor size (> 4 cm) did not affect PFS or OS [8]. Given the larger population in our study, the findings are likely more reliable and provide stronger evidence for tumor size as a prognostic factor.

The treatment of CCAC remains a major concern for clinicians. Currently, there is still no consensus on the first line therapeutic regimen for CCAC, though treatments for cervical adenocarcinoma are often referenced [22]. In our study, surgical treatment was associated with longer OS and CSS in CCAC patients. Similarly, another study analyzing treatment patterns and prognosis based on the SEER database observed a survival benefit from surgical resection in CCAC patients, particularly for patients in IB3–IIA2 and IIIC subgroups [14]. Other studies also reported a potential benefit from surgery [8]. The effectiveness of other treatments, such as radiotherapy and chemotherapy, remains unclear due to the rarity of CCAC and the predominance of surgery as the primary treatment. Liu's study found that neither adjuvant radiotherapy alone nor concurrent chemoradiation therapy (CCRT) impacted PFS or OS in CCAC patients with risk factors [8]. In our cohort, the addition of chemotherapy or radiotherapy was associated with improved OS and CSS, but these findings are observational and cannot establish causality. Therefore, while surgery remains the mainstay of treatment, the potential role of combination therapies requires further investigation in larger cohorts and ideally in prospective or randomized studies.

To our knowledge, this is the first study to develop and validate nomograms for predicting CSS and OS in CCAC patients. The nomograms demonstrated high accuracy, with C‐indices exceeding 0.8 in the validation cohort, indicating strong discriminative ability for 1‐, 3‐, and 5‐year survival. Although the calibration curves have large confidence intervals, which may be associated with limited sample size, compared with the 2018 FIGO staging system, the nomograms showed superior predictive performance. For example, the C‐index for 5‐year OS was 0.877 for the nomogram versus 0.773 for FIGO. In addition, the 5‐year OS NRI was 0.287 and the 5‐year CSS IDI was 0.154, indicating improved patient risk reclassification and overall discrimination. Decision curve analysis further confirmed that the nomograms provided greater net clinical benefit across a range of threshold probabilities. These findings suggest that the nomograms outperform FIGO staging by integrating multiple independent prognostic factors, such as age, tumor size, and histological features, which FIGO stage alone does not capture. Clinically, the nomograms can be applied to stratify patients into low‐ and high‐risk groups, guiding individualized treatment decisions—such as selecting candidates for more aggressive therapy or closer surveillance—and optimizing follow‐up intervals. Additionally, they can support physician–patient communication by providing quantitative, patient‐specific survival probabilities, enabling more informed shared decision‐making.

Despite developing a prognostic model with good predictive performance for CCAC using the SEER database, several limitations should be acknowledged. First, the study is retrospective, which may introduce selection and information biases. Second, the SEER database lacks key information on recurrence, detailed treatment regimens, and molecular or genomic profiles, limiting the inclusion of these important prognostic factors. Third, the cohort primarily represents patients from the United States, and no independent external validation cohort was included, which may constrain the generalizability of the model. Finally, the rapidly evolving landscape of CCAC treatment, including emerging therapies, may affect patient outcomes over time, suggesting that retrospective models need periodic updates to maintain clinical relevance. These limitations highlight the need for well‐designed prospective, multi‐center studies and the incorporation of additional prognostic factors, including molecular and imaging markers, to further validate and refine the model.

## 5 Conclusion

In this study, we constructed a prognostic model for predicting OS and CSS in CCAC patients based on the SEER database. The model demonstrated good predictive performance and may provide additional guidance in clinical practice by helping clinicians stratify patients according to risk, inform follow‐up schedules, and support treatment planning. Nevertheless, its clinical utility should be validated in independent, prospective cohorts, and future studies could integrate additional data (including molecular and imaging data) to further improve predictive accuracy.

## Author Contributions

**Lijun Chen:** writing – original draft, data curation, formal analysis, investigation. **Linying Liu:** writing – original draft, formal analysis, methodology, data curation, writing – review and editing. **Feishuang Lin:** software, formal analysis, methodology, visualization, validation. **Yixin Fu:** software, formal analysis, visualization, validation. **Lin Yang:** conceptualization, writing – review and editing. **Yang Sun:** conceptualization, funding acquisition, supervision, project administration, resources.

## Funding

This work was supported by Major Scientific Research Program for Young and Middle‐aged Health Professionals of Fujian Province, China (2022ZQNZD008) and Clinical Research Center for Precision Treatment of Gynecological Malignancies of Fujian Province, China (2022Y2015).

## Ethics Statement

As the clinical information and data used in this SEER study were downloaded from a public database, ethical approval and informed consent were waived.

## Consent

The authors have nothing to report.

## Conflicts of Interest

The authors declare no conflicts of interest.

## Supporting information


**Figure S1:** Flow diagram illustrating recruitment of patients.

**Figure S2:** ROC curve analysis for 1‐, 3‐, and 5‐year survival of 2018 FIGO stage. (A) OS in the training set, (B) OS in the validation set, (C) CSS in the training set, (D) CSS in validation set.

## Data Availability

Publicly available datasets were analyzed in this study. The datasets analyzed in this study are available in the SEER repository and can be obtained from: https://seer.cancer.gov/.
