# Supplementary figures and images for: Development and Validation of Prediction Models for the Prognosis of Clear Cell Adenocarcinoma of the Cervix: A Population‐Based Cohort Study
Source: Cancer Med. 2026 Jan 26;15(2):e71585. doi: 10.1002/cam4.71585 (PMC12835606; doi:10.1002/cam4.71585)

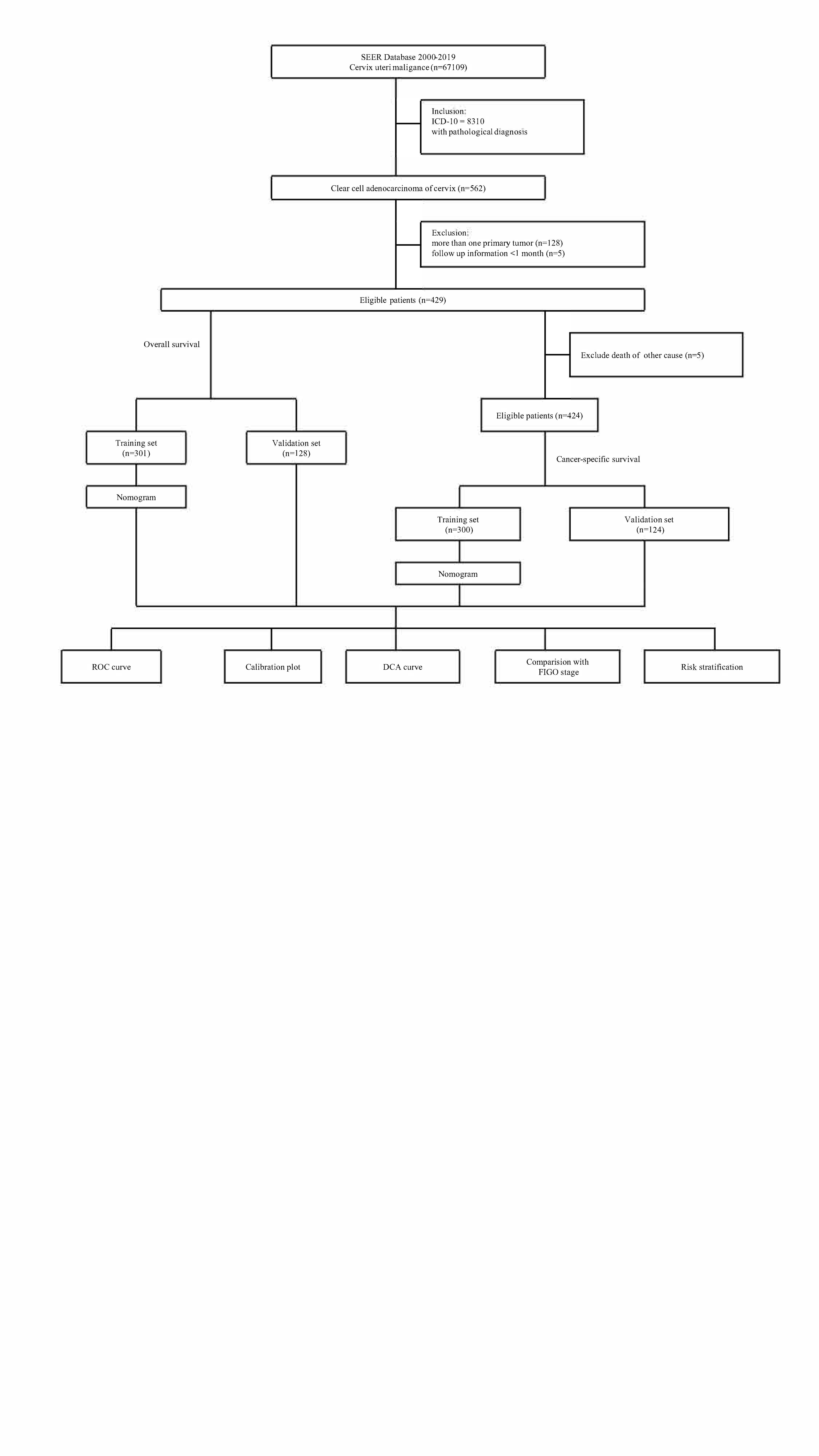

Supplement: Supplementary file 1 — Figure S1: Flow diagram illustrating recruitment of patients. [file CAM4-15-e71585-s001.jpg]

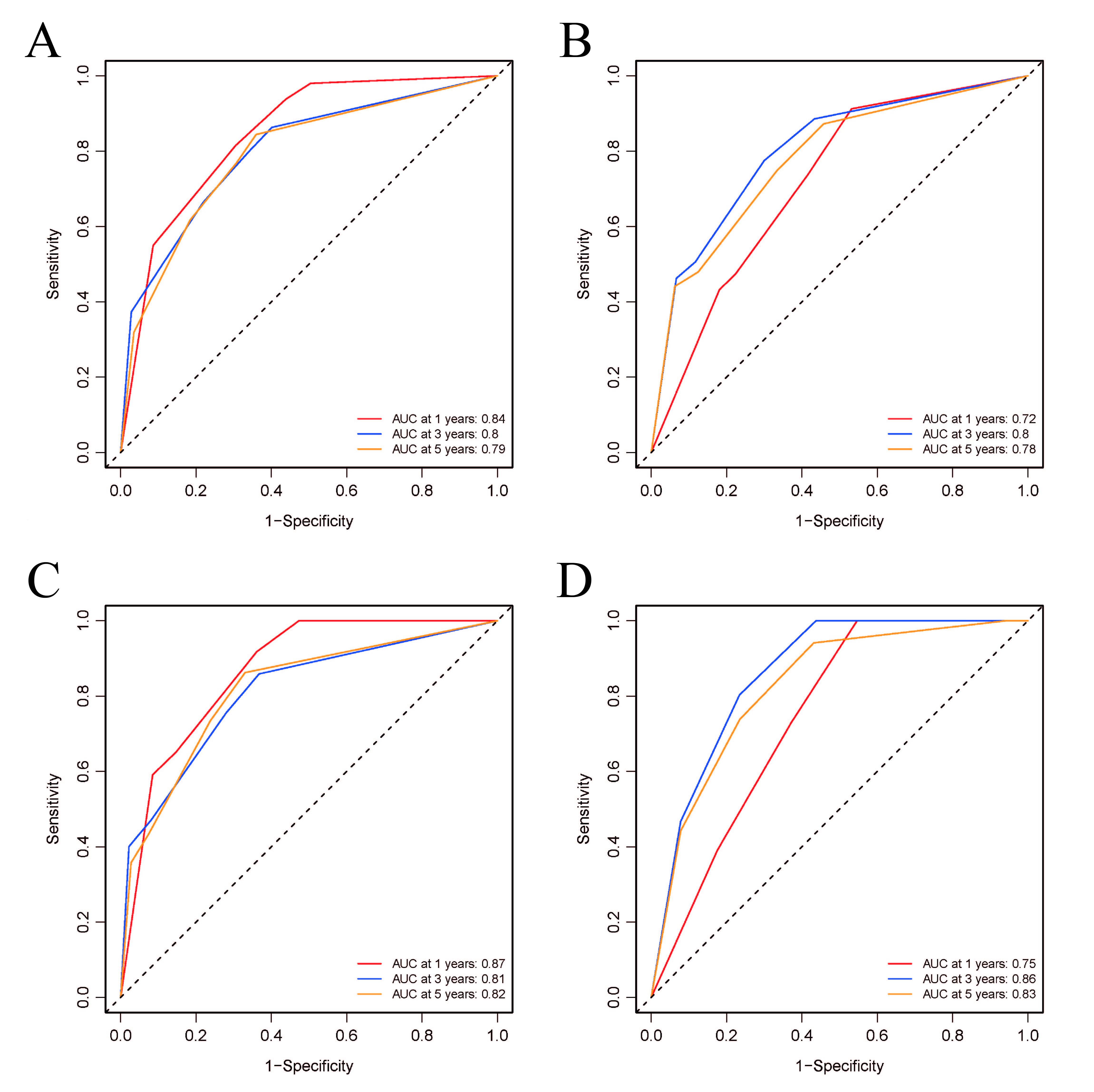

Supplement: Supplementary file 2 — Figure S2: ROC curve analysis for 1‐, 3‐, and 5‐year survival of 2018 FIGO stage. (A) OS in the training set, (B) OS in the validation set, (C) CSS in the training set, (D) CSS in validation set. [file CAM4-15-e71585-s002.jpg]
